# Toxic Action of Carbonic and Other Weak Acids on the Meningococcus

**Published:** 1918-11

**Authors:** 


***Toxic Action of Carbonic and Other Weak Acids on the Meningococcus.*** By J. A. Shaw-Mackenzie, M. D. *Journal of the Royal Army Medical Corps*, July, 1918.

It would be well, in searching for an antiseptic therapy for cerebro-spinal meningitis, to use as the basis of the antiseptic fluid in view a solution as harmless as possible to mammalian living tissues.

The faculty of conserving the vitality of such tissues with the Ringer-Locke fluid has been abundantly proved. Professor Falliberton likens this Ringer-Locke fluid very closely to the cerebrospinal fluid, though it is not of physiological origin. Therefore it could be safely used as a basis of antiseptic solutions in the therapy of the nervous system.

After researches as to the toxic action of copper compounds of amino-acids, Lieutenant-Colonel Mervyn Gordon and Major Hine found that the meningococcus did not survive in concentration of copper alanine 1 in 1.000 in sodium chloride solution on 20 minutes exposure. Since a short time is unquestionably essential for purposes of intrathecal or naso pharingeal treatment, the concentration is not to be especially selected, the meningococcus surviving in less than a 20 minutes' exposure.

The observation that the meningococcus did not survive in the Ringer-Locke's fluid modified led to the belief that this fluid would possess the same bacterial properties for irrigations and intrathecal use.

Flexner and Shearer conclude that physiological solutions of sodium chloride have a destructive action on freshly isolated meningococci after long exposure. Later experiments with sodium bicarbonate proved that a 0.02 percentage of $NaHCO_3$ in Ringer-Locke fluid has less toxic effect than one per cent. This could hardly be attribued to the effect of its alkalinity.

Acetic acid : A toxic result was ultimately obtained by dissolving 0.2 cubic centimeter suspension to ten cubic centimeters solution of acetic acid, of strength respectively one per cent., 1/1000, 1 in 10,000 in 0.9 per cent. sodium chloride solution. The 1/10,000 solution proved fatal to the meningococcus after 5 minutes' exposure.

But Doctor Locke composed a solution which presents particular and marked interest. This solution contains carbonic acid, and is easily sterilized and assimilable for the mammalian heart; a serum through which a current of carbonic acid has been passed exerts an increased and more rapid toxic action on the meningococcus. The method depends on the conversion of sodium carbonate by sulphuric acid into sodium sulphate, sodium bicarbonate and the percentage of $CO_2$ required. Of course it should be remembered that carbonic acid stands in a special physiological position. Increased percentage of it might in physiological fluids exert a specific inhibitory effect on the vital activity of micro-organisms in addition to its effect in increasing hydrogenion concentration.

Lactic acid proved to have a definite toxic effect, for the meningococcus did not survive after 5 minutes' exposure.

It is yet hard to formulate any opinion on the bactericidal action of hydrogenion concentration. Experiments have not been conclusive enough so far.

